# Determination of Flavonoid and Proanthocyanidin Profile of Hungarian Sour Cherry

**DOI:** 10.3390/molecules23123278

**Published:** 2018-12-11

**Authors:** Andrea Nemes, Erzsébet Szőllősi, László Stündl, Attila Biró, Judit Rita Homoki, Mária Magdolna Szarvas, Péter Balogh, Zoltán Cziáky, Judit Remenyik

**Affiliations:** 1 Institute of Food Technology, University of Debrecen, H-4032 Debrecen, Hungary; nemes.andrea@agr.unideb.hu (A.N.); szzsoka83@gmail.com (E.S.); stundl@agr.unideb.hu (L.S.); attila.biro88@gmail.com (A.B.); homoki.judit@agr.unideb.hu (J.R.H.); sebestyen.magdolna@agr.unideb.hu (M.M.S.); 2 Faculty of Economics and Business, Institute of Sectoral Economics and Methodology, Department of Research Methodology and Statistics, H-4032 Debrecen, Hungary; balogh.peter@econ.unideb.hu; 3 Agricultural and Molecular Research and Service Institute, University of Nyíregyháza, H-4400 Nyíregyháza, Hungary; cziaky.zoltan@nye.hu

**Keywords:** sour cherry, anthocyanins, extractable polyphenols, non-extractable polyphenols

## Abstract

Hungarian sour cherries (SC) are excellent source of anthocyanin (concentrations (100–300 mg in 100 g fresh fruit) and melatonin (0.15 mg in 100 g fresh fruit), but other flavonoid derivatives also can be isolated by aqueous alcoholic extraction. We have developed a new process for extracting non-extractable procyanidines bound to the membrane, proteins, and fibers. These compounds were seperated with UHPLC-MS methods, and the structure of individual components were identified on the basis of their mass fragmentation spectra. The antioxidant capacity of soluble and non-soluble antioxidants were measured with ferric reducing antioxidant power (FRAP), 1,1-diphenyl-2-picrylhydrazyl radical scavenging activity (DPPH), trolox equivalent antioxidant capacity (TEAC) assays, and compared to the new measurement methods of water-soluble antioxidant capacity (ACW), lipid-soluble antioxidant capacity (ACL). Furthermore, total phenolic content (TPC) and total procyanidin content (PAC) were determinated. As a result of our investigation, we found that the solvent combination, where in the first step is water–ethanol (1:1), then 100% ethanol were suitable for the extraction of the extractable antioxidants. However, the chemiluminescence method that is based on the elimination of the superoxide radical is more accurate than other colorimetric methods which measure antioxidant capacity.

## 1. Introduction

Sour cherry (*Prunus cerasus* L.) belongs to the family of Rosaceae, subfamily Prunoideae, to the genus Prunus, subgenus Cerasus. This is the hybrid that is produced by crosses between sweet cherry (*Prunus avium* (L.) L.) and European dwarf cherry (*Prunus fruticosa*) [1]. According to the FAOSTAT, world production of sour cherries in 2016 was 1,378,216 tons. The largest producers of cherries are Russia, Poland, Turkey, and the USA. Hungary is only the eighth, with nearly 70 thousand tons. Furthermore, the Hungarian cultivation of sour cherry has several centuries of history. Because of the geographic and climatic conditions of Hungary and the long breeding work, the Hungarian cultivar assortment and their varieties wiht outstanding nutritional parameters were developed, that are unique in the world. The popularity of the Hungarian varieties is shown by the fact that a Hungarian cultivar ‘Újfehértói fürtös’ was introduced into the United States in 1984 (in Michigan, Utah, and Wisconsin), that is marketed under the name Balaton and is regarded as a super food [2]. In recent years, clinical trials have shown the positive physiological effects of various components that accumulate in the cherry. Different classes of flavonoids have been shown to increase the expression of the gene encoding the γ-glutamylcysteine synthetase catalytic subunit, a protein reported to be the rate-limiting step in GSH synthesis [3,4]. Cy3G has cytoprotective effects, so it protects cells such as aortic endothelial cells (EC) by reducing hydrogen peroxide (H_2_O_2_)-induced oxidative stress in vitro and in vivo [5,6,7]. Youdim et al. (2000) presented for the first time that vascular endothelial cells can incorporate anthocyanins (mainly cyanidin-3-glucoside) into the membrane and cytosol, and the incorporation of anthocyanins by the EC significantly enhanced their resistance to the damaging effects of reactive oxygen species (ROS) [8].

Moreover, flavonoids have anti-oxidative, anti-inflammatory, anti-mutagenic, and anti-carcinogenic properties coupled with their capacity to modulate key cellular enzyme function. Lipoxygenase, xanthine oxidase, and NADPH oxidase enzymes in EC are activated during inflammatory processes. The activity of the NADPH oxidase enzyme can cause endothelial dysfunction in two ways. The activity of the NADPH oxidase enzyme produces oxygen-containing free radicals. The first is that the resulting superoxide anion (O2 •−) directly affects the function of the endothelial nitric oxide synthase (eNOS) enzymes by utilizing the cofactor needed for NO synthesis. It follows that the functioning of the eNOS enzyme is disturbed. The second is that O2 •− reacts with NO and connects to peroxinite. Peroxynitrite damages membrane proteins therefore it causes the disruption of mitochondrial electron transport chain [9].

Anthocyanins and some flavone and flavan-3-ol compounds may contribute to the prevention of hypertension [10]. Furthermore, it was demonstrated that the dietary anthocyanin Cy3G acts as a natural activator of eNOS in EC [11]. It has also been known that many extracellular agents (free fatty acids, H2O2, TNF-α) contribute to insulin resistance [12].

Furthermore, Guo et al. (2008) investigated the effect of Cy3G on H_2_O_2_- and TNF-α-induced insulin resistance on the 3T3-L1 adipocyte cell culture. Based on their measurements, it has been demonstrated that Cy3G protects adipocytes by inhibiting the kinase activity of the c-Jun NH2 terminal kinases, so the phosphorylation of the insulin receptor protein (IRS1) occurs via tyrosine [13].

The most significant antioxidant compounds in SC are phenol carboxylic acids (hydroxycinnamic acid, chlorogenic acid, neochlorogenic acid, p-coumaroylquinic acid) [14,15], flavanols (catechin, epicatechin, epigallocatechin, gallocatechin), and derivatives [16,17]. The accumulation of melatonin is also significant [18,19]. Polyphenol content in sour cherries and its health effect have been widely studied, for the extractable polyphenols only. Antioxidant capacity is usually measured in food extracts with different combination of organic solvents (e.g., methanol, ethanol, acetone) and water, but these usually do not result complete extraction of antioxidant compounds. This is mainly problem in the case of phenolics compounds, because these compounds are extracted by organic solvents, which probably leaves behind significant other phenolics existing in bound form. Usually, just the amount of extractable fractions were analyzed, the non-extractable compounds (in the solid residue) were ignored and associated with cell wall matrix. Nevertheless, unextractable phenolics from plant foods and their role in health benefits have become increasingly important [14,15,16].

These non-extractable phenolic compounds are considered to contribute more beneficial effects (gastrointestinal health, cancer, cardiovascular disease) because after gastrointestinal digestion they remain undegraded, and are absorbed into blood plasma after being released by intestinal microflora fermentation [17,18].

Phenolics can be classified as soluble and insoluble-bound form. Thus, the polyphenols that can be extracted from foods with aqueous-organic solvent, called extractable polyphenols (EPP). However, a significant fraction of polyphenols remains in the residue after the extraction; the so-called non-extractable polyphenols (NEPP) [19].

The solid residue contains macromolecules (e.g., high-molecular-weight proanthocyanidins) and single phenolic compounds (e.g., phenolic acids, associated with macromolecules) mainly polysaccharide constituents of dietary fibre and protein. NEPP are generally not included in polyphenol analysis, however, the NEPP content may be much higher than the EPP fraction. With regards to their chemical nature, NEPP mainly include polyphenols such as proanthocyanidins, other flavonoids, phenolic acids, and hydrolysable tannins. Accordingly, the NEPP usually were divided into two groups, hydrolysable tannins and non-extractable proanthocyanidins (NEPA) [18,20].

Acid and alkaline hydrolysis are the most common chemical methods used to extract the NEPP and recently, many other new methods such as enzymatic hydrolysis has been employed for better release of NEPP from cell wall matrices [14,21,22,23].

Plants contain various chemical compounds and the antioxidant effectiveness is determined by many factors (the heterogeneity and heterophasic nature of the system, the type of lipid substrate, including its physicochemical state and degree of unsaturation, the types of initiators (notably transition metals), other components, and their possible interaction).

Because of these, for assessment of antioxidant potential of endogenous compounds, single assay methods are not sufficient.

Several in vitro methods exist to measure the total antioxidant capacity. The different antioxidant assays differ in terms of assay principle and experimental conditions. Depending on what kind of reaction is involved, these assays can be classified into two groups: assays based on hydrogen atom transfer (HAT) reactions and assays based on electron transfer (ET) [24].

The most widely used procedures are FRAP (ferric reducing antioxidant power), ABTS (2,2′-Azino-bis(3-ethylbenzothiazoline-6-sulfonic acid)) or TEAC (trolox equivalent antioxidant capacity), DPPH (2,2-diphenyl-1-picrylhydrazyl), and oxygen radical absorbance capacity (ORAC). The FRAP, TEAC, and DPPH methods belong to ET methods. Unfortunately, however, they have some disadvanteges and limitations. The main disadvantage of FRAP method is that the measured reducing capacity does not necessarily reflect antioxidant activity. Since the method does not include an oxidisable substrate, no information is provided on the protective properties of antioxidants [25]. DPPH assay is limited because DPPH radicals interact with other radicals (alkyl), and the time response curve to reach the steady state is not linear with different ratios of antioxidant/DPPH [26,27]. The TEAC assay also has several limitations. The ability of an antioxidant to scavenge the artificial ABTS radical may not reflect the antioxidant activity due to other mechanisms effective in complex food lipids or physiologically relevant substrates, including metal chelation and effects of antioxidant partitioning among phases of different polarities [25]. The biological and physiological functions of antioxidants are wide-ranging. The identification and quantitative determination of antioxidants, characterization of antioxidant capacity, and the evaluation of interactions between different food matrices can only be done by examining the entire antioxidant system. In order for it to be compare or examine foods with the same or different matrices, an extraction process have to develop that can efficiently obtaining the active compounds, where the componunds retain their chemical composition. Since it is important to identificate the antioxidant compounds, and to determine the antioxidant capacity of the components as accurately as possible. The choice of the right method is also a goal because oxidative stress is well known and studied, but there is antioxidative stress too.

In our opinion, PLC technique (HAT method) can be the best choice to determine the antioxidant capacity, since this method is based on the photo-induced autooxidation inhibition of luminol by antioxidants, mediated from the radical O_2_^•−^, which can be found in human body, and is suitable to measure the radical scavenging properties of single antioxidants as well as more complex systems in the nanomolar range.

This study aims at investigating the differences between extraction processes. The first was commonly used combination of methanol, acetone, and water by Saura-Calixto and Goñi [28], and the second was an ethanol extraction that is used by the food and pharmaceutical industry too. During the experiment, not only were the extractable fractions analyzed, but also non-extractable compounds in the solid residue.

## 2. Results and Discussion

### 2.1. Extractable Antioxidant Compounds of Sour Cherry

Extracts (1/A + 1/B and 2/A + 2/B), were obtained by the two extraction methods, and analyzed with UHPLC-MS (Table 1). It can be seen that the solution combination 1 and 2 also extracted flavonoids and phenolic components. Among the flavonoids, anthocyanin-glucosides [29] and flavanol-*O*-glycosides [30] occur in large quantitiesin the sour cherry. The combination of solvent 1 has proved to be more effective mainly in the extraction of procyanidin C isomer.

### 2.2. Main Anthocyanin Compounds of Sour Cherry

**Figure 1:**
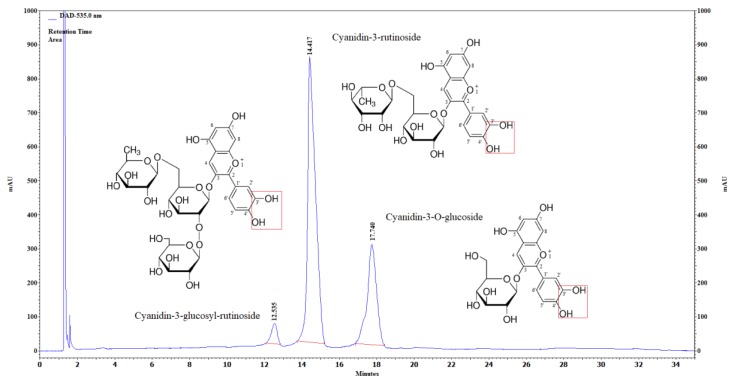
UHPLC chromatogram of sour cherry at 535 nm. Confirmed by standard.

The main anthocyanin components in the ‘Újfehértói fürtös’ variety (Figure 1.) were the cyanidin-3-*O*-glucosyl-rutinoside, (2 mg/100 g), cyanidin-3-*O*-rutinoside, (183 mg/100 g) and cyanidin-3-*O*-monoglucoside (4.29 mg/100 g).

The antioxidant capacity of these components is determined by OH groups at positions C3′ and C4′ on the chalcone.

### 2.3. Main Flavonoid and Phenolic Compounds of Sour Cherry

**Figure 2:**
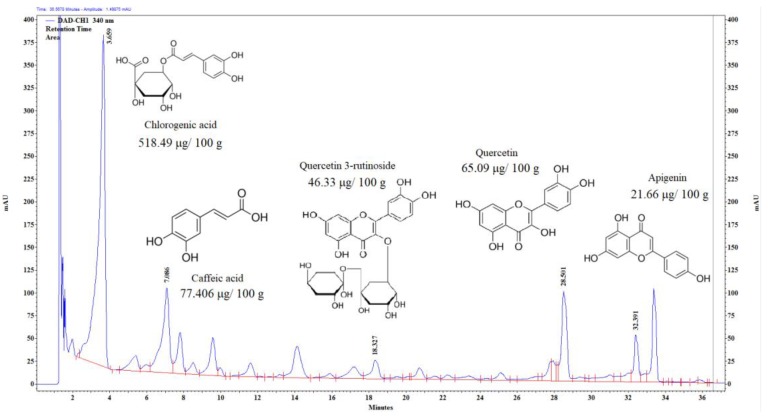
UHPLC chromatogram of sour cherry at 340 nm. Confirmed by standard.

Quercetin, quercetin-3 rutinoside and apigenin (flavonoids) occur in abundant quantities in sour cherry (Figure 2.). These are precursor compounds in the biosynthesis of anthocyanins. There are other phenolic compounds like chlorogenic and caffeic acid in high concentrations in this fruit. The antioxidant activity of these compounds is also high.

### 2.4. Total Procyanidin Content (PAC) of Sour Cherry and Sour Cherry Residues

Several studies have been reported that procyanidins have strong protective properties regarding oxidative damage, microbial infection, prevention of colon cancer, and prevention of cardiovascular disease [37,38,39,40]. However, the degree of polymerization (DP) of procyanidins may be highly influential and determine these effects [41,42,43]. In the gastrointestinal tract, procyanidin monomers, dimers, and trimers are absorbed into the blood system to a much larger extent than larger oligomers and polymers [44,45]. The DP value of sour cherry procyanidins is lower than 4, indicating the relatively high levels of better absorbable short-chain procyanidin species [46], so sour cherry is an exceptional source of short-chain procyanidins, and a major food ingredient.

**Figure 3:**
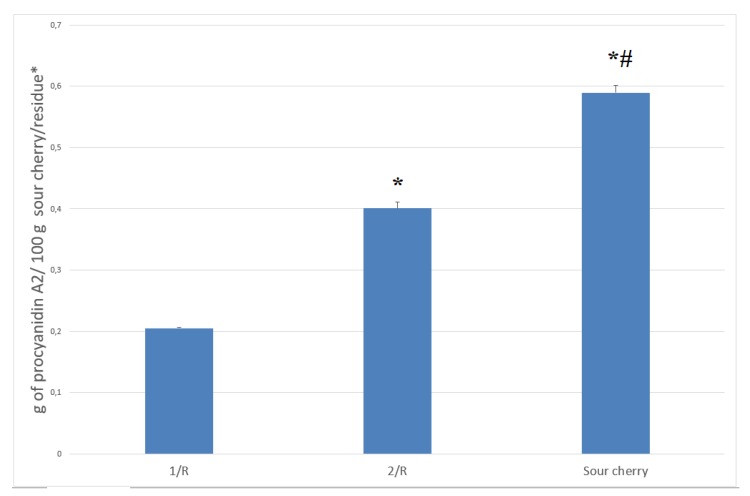
The total procyanidin content of sour cherry and sour cherry residues. * Units for different type of samples: sour cherry sample: g of procyanidin A2/100 g fresh weight sour cherry; residues: g of procyanidin A2/100 g dried residue. Abbreviations: 1/R: residue of ‘solvent combination 1′; 2/R: residue of ‘solvent combination 2′. * indicates significant difference (*p* < 0.05) from the 1/R. # indicates significant difference (*p* < 0.05) between the 2/R and the sour cherry.

Measurably higher amounts of mono-, di-, and trimer procyanidins were extracted with the solvent combination 1, since fewer remained in the residue 1/R, however, the total amount of procyanidins could be not extracted (Figure 3.).

### 2.5. Identification of Cinconain I

**Figure 4:**
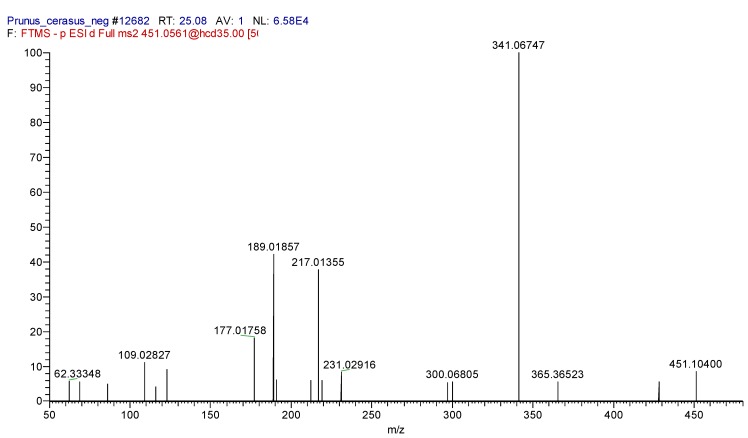
ESI-MS2 spectrum of Cinchonain I diastereoisomer at retention time 25.51.

As you can see in the Table 1, the peaks for Cinchonain I isomers are 19.41, 21.81, 22.81, and 25.51 min. with [M − H]^−^ ions at *m*/*z* 451.10291 were identified as distereomers of Cinchonain I on the bases of their exact molecular mass, isotopic pattern, and fragmentation. The characteristic fragment ions are 451.10400, 341.06747, and 217.0155 (Figure 4.).

### 2.6. Extractable Antioxidant Capacity of Sour Cherry Extracts

**Figure 5:**
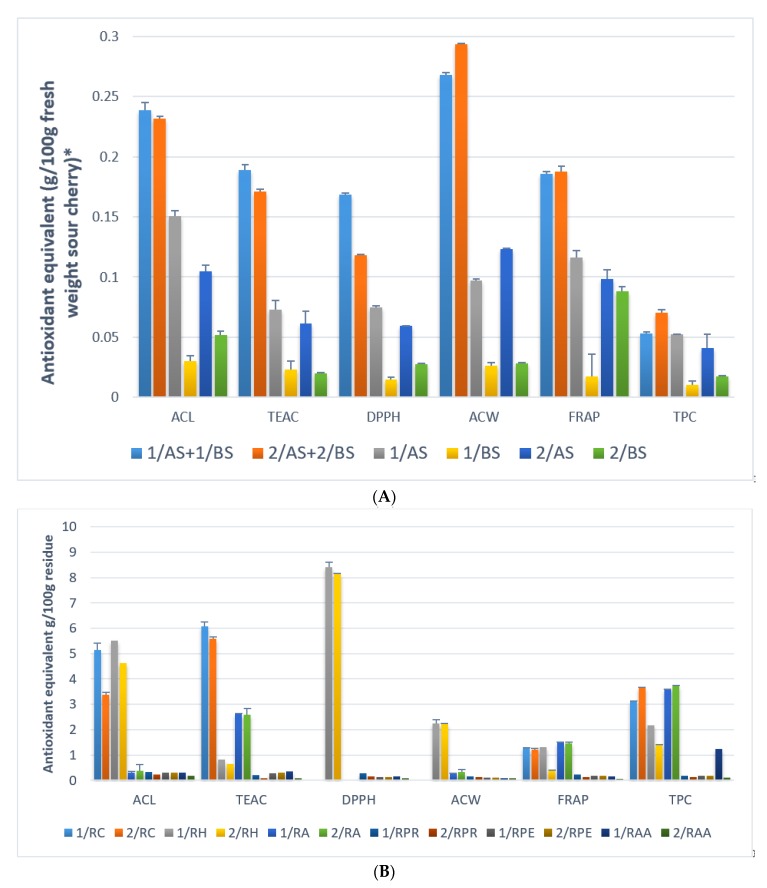
ESI-MS2 spectrum of procyanidin B. (**A**) Comparison of the extractable antioxidant capacity of sour cherry-extracts and the totalphenolic content. * Units for different measurement methods: ACL: Trolox equivalent g/100 g; TEAC: Trolox equivalent g/100 g; DPPH: Trolox equivalent g/100 g; ACW: Ascorbic acid g/100 g; FRAP: Ascorbic acid g/100 g; TPC: Gallic acid g/100 g. Abbreviations: 1/AS: evaporated ethanol:water extract; 1/BS: evaporated ethanol extract after ethanol:water extraction; 1/AS + 1/BS: 1/AS and 1/BS extracts evaporated together; 2/AS: evaporated acidic methanol:water extract; 2/BS: evaporated acetone:water extract after methanol:water extraction; 2/AS + 2/BS: 2/AS and 2/BS extracts evaporated together. The table form (Supplement 1) with the results of the statistical analysis can be found in the Supplemets secyion. (**B**) Comparison of the non-extractable antioxidant capacity of sour cherry residue extracts and the totalphenolic content. * Units for different measurement methods: ACL: Trolox equivalent g/100 g; TEAC: Trolox equivalent g/100 g; DPPH: Trolox equivalent g/100 g; ACW: Ascorbic acid g/100 g; FRAP: Ascorbic acid g/100 g; TPC: Gallic acid g/100 g. Abbreviations: 1/R: residue of ‘solvent combination 1′; 1/RC: supernatant from the ‘extraction of hydrolysable tannins’ of 1/R; 1/RH: supernatant from the ‘extraction of condensed tannins’ of 1/R; 1/RA: supernatant from the ‘alkaline hydrolysis’ of 1/R; 1/RPR: supernatant from the ‘protease hydrolysis’ of 1/R; 1/RPE: supernatant from the ‘pectinase hydrolysis’ of 1/R; 1/RAA: supernatant from the ‘α-amilase hydrolysis’ of 1/R; 2/R: residue of ‘solvent combination 2′; 2/RC: supernatant from the ‘extraction of hydrolysable tannins’ of 2/R; 2/RH: supernatant from the ‘extraction of condensed tannins’ of 2/R; 2/RA: supernatant from the ‘alkaline hydrolysis’ of 2/R; 2/RPR: supernatant from the ‘protease hydrolysis’ of 2/R; 2/RPE: supernatant from the ‘pectinase hydrolysis’ of 2/R; 2/RAA: supernatant from the ‘α-amilase hydrolysis’ of 2/R; The table form (Supplement 2) with the results of the statistical analysis can be found in the Supplemets chapter.

The antioxidant activites were compared in case of solvent combination 1 and 2, using the well-known measuring methods FRAP, DPPH, TEAC, ACL, ACW, and TPC (Figure 5A.).

Our results show that the amount of antioxidant compounds extracted by these two methods is not significantly different. The aqueous–alcoholic mixture is more advantageous for the processing industry and is sufficient to extract the most important compounds of the sour cherry (anthocyanins, procyanidines, phenolic components, and flavonoids). These compounds slightly soluble in water, but ethanol is a good solvent for them. Because of the polarity of 1/A mixture, it is also suitable for extraction of low molecular weight organic acids, which provides pH 3 in which glycolized anthocyanins maintain their chemical structure (more acid is not required.). All the other antioxidants can be extracted with ethanol (1/B).

UHPLC-MS measurements show (Table 1) that combinations of 1/A or 1/B and 2/A or 2/B are only partially applicable to the extraction of bioactive compounds, because all sour cherry extracts (1/AS, 1/BS, 2/AS, 2/BS) include ‘anthocyanin and procyanidins’, ‘flavonoids’, and ‘other polyphenols’. The difference is only the amount of extracted compounds, that depends on solubility. It is therefore not surprising that methods based on the measurement of water-soluble polar components can measure a lower antioxidant concentration.

The used assays (except for ACL, ACW) are colorimetric methods based on complexometry, and have many disadvantages. The most significant drawback of the FRAP method is that it is only suitable for measuring water soluble components. In addition, not all antioxidants are able to reduce Fe^3+^, antioxidants that act by H atom transfer are not detected [47,48,49]. During the application of the TEAC method, the problem is that the reaction of ABTS+• with the antioxidant compounds is strongly time-dependent, so components with ‘slow kinetic’ do not react with the radical in time [47]. DPPH is mainly used to measure the ability of polyphenols to transfer labile H atoms to radicals [50].

Obviously, the determination of antioxidant capacity of antioxidant compounds, that are extracted with solvent combination 1 and 2 by chemiluminescence technique seem to be the most appropriate. This method is suitable for selectively determining the concentration of water-soluble and lipid-soluble antioxidants. It can be used for antioxidants that act H atom transfer and electron transfer, because both groups react with the superoxide anion, which is one of the most important radicals in the living organisms [51,52].

### 2.7. Capacity of Non-Extractable Antioxidants of Sour Cherry Extracts

The enzymatic digestion not resulted significant antioxidant activity (Figure 5B.). The explanation is that sour cherry does not contain large amounts of starch, so the α-amylase hydrolisis is not relevant. It does not contain large amounts of protein, so hydrolysis with protease is not important either. The use of pectinase is also more important for apple crops.

Significant amounts of antioxidant fragments were obtained in the case of the alkaline treatment, but mainly acidic hydrolysis. The results obtained using the HCl butanol extractant are not significantly different. Low molecular weight phenolic derivatives resulting from the degradation of polyphenols may be responsible for the apparently high antioxidant activity.

Proantocianines are considered as non-extractable components but some of them can be recovered with a solvent combination 1 and 2, as can be seen from the Table 1. However, non-extractable proantocianidines can not be identified in the hydrolysates.

The amount of antioxidants that extracted from the sour cherry and obtained from hydrolysis from the remaining residues were similar in case of the solvent combination (Figure 5A,B). Thus, the acidification (HCl) and the methanol–acetone–water solvent combination that was used by Saura-Calixto and Goñi [28] did not extract significantly higher amounts of antioxidant compounds. Consequently, it is not necessary to use HCl, and the methanol can be replaced by etanol in the first step, furthermore the acetone water combination can be replaced by 100% ethanol in the second step.

## 3. Experimental

### 3.1. Plant Material

Stoned, frozen sour cherry (’Újfehértói fürtös’ variety) was bougth from Mirelite Mirsa Zrt (Albertirsa, Hungary) in 2017. Fruit samples were frozen (−20 °C) and stored in dark.

### 3.2. Chemicals and Reagents

Ethanol, acetic acid, and Folin–Ciocalteu’s reagent were purchased from VWR (Randore, PA, USA). Hydrochloric acid and 1-Butanol were obtained from Merck (Damstadt, Germany). Methanol, acetone, sulfuric acid, 2, 4, 6-Tris(2-pyridyl)-s-triazine (TPTZ), 2,2-Diphenyl-1-picrylhydrazyl (DPPH), 2,2′-azino-bis(3-ethylbenzothiazoline-6-sulforic acid) (ABTS), 4-(dimethylamino)cinnamaldehyde (DMAC), iron(III) chloride hexahydrate, sodium acetate trihydrate, potassium persulfate, procyanidin A2, sodium carbonate anhydrous, ascirbic acid, (±)-6-Hydroxy-2,5,7,8-tetramethylchromane-2-carboxylic acid (Trolox), gallic acid, sodium hydroxide, antioxidant standards, and the enzymes (protease, α-amylase) were purchased from Sigma-Aldrich (St. Louis, MO, USA). Pectinex XXL enzyme was obtained from Novozymes (Bagsværd, Denmark). Dulbecco’s phosphate-buffered saline (DPBS) were obtained from iBioTech (Szigetszentmiklós, Hungary). ACL and ACW kits were obtained from Greenlab (Budapest, Hungary). In our experiments, all the reagents were analytical grade. HPLC-grade methanol and formic acid were purchased from Fisher Scientific (Hampton, NH, USA).

### 3.3. Extraction of Extractable Antioxidants

The sour cherry was defrosted and homogenized. Then two different solvents were used to extract antioxidant compounds. Each extraction was performed parallel three times.

#### 3.3.1. Extraction with the Mixture of Ethanol and Water (Solvent Combination 1)

200 g of sample was extracted with 150 mL ethanol (96%) and 150 mL distilled water for 2 h (1/A). The samples were centrifuged (Eppendorf Cetrifuge 5810R)) for 15 min at 4000 rpm and the supernatant was recovered.

150 mL ethanol (96%) was added to the residue, and the mixture was mixed for 2 h (1/B). After centrifugation (15 min, 4000 rpm), the supernatant was recovered.

**Figure 6:**
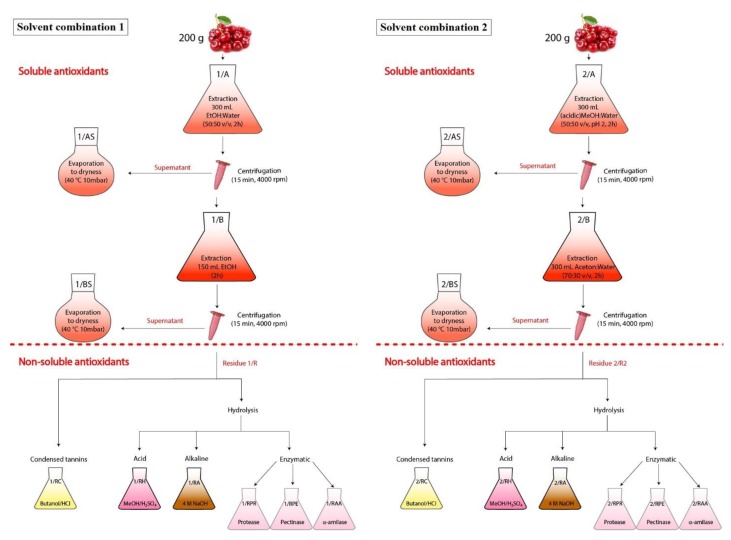
Scheme of the extraction of antioxidants from sour cherry. Abbreviations: 1/AS: evaporated ethanol:water extract; 1/BS: evaporated ethanol extract after ethanol:water extraction; 1/AS + 1/BS: 1/AS and 1/BS extracts evaporated together; 2/AS: evaporated acidic methanol:water extract; 2/BS: evaporated acetone:water extract after methanol:water extraction; 2/AS + 2/BS: 2/AS and 2/BS extracts evaporated together; 1/R: residue of ‘solvent combination 1′; 1/RC: supernatant from the ‘extraction of hydrolysable tannins’ of 1/R; 1/RH: supernatant from the ‘extraction of condensed tannins’ of 1/R; 1/RA: supernatant from the ‘alkaline hydrolysis’ of 1/R; 1/RPR: supernatant from the ‘protease hydrolysis’ of 1/R; 1/RPE: supernatant from the ‘pectinase hydrolysis’ of 1/R; 1/RAA: supernatant from the ‘α-amilase hydrolysis’ of 1/R; 2/R: residue of ‘solvent combination 2′; 2/RC: supernatant from the ‘extraction of hydrolysable tannins’ of 2/R; 2/RH: supernatant from the ‘extraction of condensed tannins’ of 2/R; 2/RA: supernatant from the ‘alkaline hydrolysis’ of 2/R; 2/RPR: supernatant from the ‘protease hydrolysis’ of 2/R; 2/RPE: supernatant from the ‘pectinase hydrolysis’ of 2/R; 2/RAA: supernatant from the ‘α-amilase hydrolysis’ of 2/R.

For the first time, supernatants from 1/A and 1/B were evaporated together (1/AS + 1/BS), but in the second case separately (1/AS and 1/BS). Evaporation was performed at 40 °C, 10 mbar (Figure 6).

The residue (1/R) was lyophilised (ScanVac CoolSafe 55-4 Pro lyophilizer), homogenized (Gorenje SMK 150 B coffee grinder), and stored in freezer (−20 °C) before use.

#### 3.3.2. Extraction of Extractable Antioxidants According to Saura-Calixtoa and Goñi [28] (Solvent Combination 2)

200 g of sample was extracted with 300 mL acidic methanol/water/HCl (50:50; pH 2) were added (2/A) for 2 h, then centrifuged (15 min, 4000 rpm) and the supernatant was recovered.

300 mL acetone/water (70:30, *v/v*) was added to the residue, and the mixture was mixed for 2 h (2/B). After centrifugation (15 min, 4000 rpm), the supernatant was recovered [28].

The extraction was performed twice. For the first time, supernatants from 2/A and 2/B were evaporated together (2/AS + 2/BS), but in the second case separately (2/AS and 2/BS). Evaporation was performed at 40 °C, 10 mbar (Figure 6).

The residue (2/R) was lyophilised, homogenized, and stored in freezer (−20 °C) before use.

#### 3.3.3. Preparation of Extracts from UHPLC

The purification of anthocyanins, a simple fractionation of sour cherry extracts (1/A + 1/B and 2/A + 2/B) was performed using preconditioned Supelclean ENVI-18 SPE tubes [53]. The tubes were conditioned with 5 mL MeOH then with 5 mL H_2_O and finally 1 mL of fruit sample was applied. The anthocyanins were eluted with methanol:water; 80:20. Solvent was evaporated at 40 °C with Heidolph Hei-VAP Value rotary evaporator (Schwabach, Germany).

### 3.4. Acid Hydrolysis

#### 3.4.1. Extraction of Hydrolysable Tannins

10–10 mg dried sour cherry residue powder from the two extractions were subjected to hydrolysis with 2 mL methanol and 200 µL sulphuric acid for 20 h at 85 °C. Samples are then centrifuged (2500 *g*, 10 min) and supernatants recovered. The residues was washed with 2–2 mL distilled water two times [54]. The supernatant from 1/R residue is 1/RH, and the extract from 2/R residue is 2/RH.

#### 3.4.2. Extraction of Condensed Tannins

10–10 mg dried sour cherry residue powder from the two extractions were treated with 3 mL HCl/butanol (5:95) and 100 µL FeCl_3_ (2 wt %) at 100 °C for 3 h. After centrifugation (2500 *g*, 10 min), the supernatant was recovered. The residues were washed with 2–2 mL HCl/butanol (5:95) two times. The supernatant from 1/R residue is 1/RC, and the extract from 2/R residue is 2/RC [55,56].

### 3.5. Alkaline Hydrolysis

0.1 g dried sour cherry residue powder from the two extractions were treated with 5 mL NaOH (4 mM) at 25 °C for 1 h. After centrifugation (4000 rpm, 10 min), the supernatant was recovered. The supernatant from 1/R residue is 1/RA, and the hydrolysate from 2/R residue is 2/RA [23].

### 3.6. Enzymatic Hydrolysis

#### 3.6.1. Enzymatic Hydrolysis with Protease

5 mL DPBS and 10 μL tyrosine (50 mg/mL; 3550 tyrosine units/mL) was added to 0.1 g dried sour cherry residue powder from the two extractions. The samples were incubated at 60 °C for 1 h. To stop the enzymatic hydrolisis, the hydrolysates were placed in a water bath at 100 °C for 10 min. After centrifugation (5 min, 4000 rpm), the supernatant was recovered. The hydrolysate from 1/R residue is 1/RPR, and the supernatant from 2/R residue is 2/RPR.

#### 3.6.2. Enzymatic Hydrolysis with Pectinase

5 mL DPBS and 10 μL pectinase (Pectinex XXL) was added to 0.1 g dried sour cherry residue powder from the two extractions. The samples were incubated at room temperature for 1 h. To stop the enzymatic hydrolisis, the hydrolysates were placed in a water bath at 100 °C for 5 min. After centrifugation (5 min, 4000 rpm), the supernatant was recovered. The supernatant from 1/R residue is 1/RPE, and the hydrolysate from 2/R residue is 2/RPE [22].

#### 3.6.3. Enzymatic Hydrolysis with α-amilase

5 mL DPBS and 10 μL α-amilase (3000 Units/mL) was added to 0.1 g dried sour cherry residue powder from the two extractions. The samples were incubated at 37 °C for 10 min. To stop the enzymatic hydrolisis, the hydrolysates were placed in a water bath at 100 °C for 20 min. After centrifugation (5 min, 4000 rpm), the supernatant was recovered. The supernatant from 1/R residue is 1/RAA, and the hydrolysate from 2/R residue is 2/RAA [23].

### 3.7. Determination of Total Phenolic Content (TPC)

Total phenolics in all extracts were determined with the Folin–Ciocalteu assay [57] with minor modifications. 10 μL of appropriately diluted extracts, standard gallic acid solutions (50, 100, 200, 400, 800, and 1600 μg/mL) or water (blank) was mixed with 190 μL of distilled water in a well of a 96-well plate; 25 μL of Folin–Ciocalteu reagent solution was then added. After 6 min, 75 μL of 7% Na_2_CO_3_ was added. The mixture was shaken gently and incubated in the pre-heated chamber (50 °C) for 10 min, and its absorbance was measured at 765 nm, using the microplate reader (SPECTROstar^®Nano^, BMG Labtech, Ortenberg, Germany). TPC was expressed as milligrams of gallic acid equivalents (mg GAE/100 g fresh weight sour cherry/dried residue). In some cases, the extract was too dilute, so 100 μL of sample and 100 μL of H_2_O were mixed in a well.

### 3.8. Determination of Total Procyanidin Content (PAC)

Total procyanidin content was measured using the method of Prior et al. [58].

Dried powder of residuals and sour cherry were weighed (500 mg) into a 50 mL conical tube. 20 mL extraction solution (acetone/deionized water/acetic acid 75:24.5:0.5) was added to the samples. The samples were vortexed for 30 s followed by sonication for 1 h at room temperature. After centrifugation (4000 rpm, 10 min), the supernatant was collected for analysis.

70 µL of 96% ethanol for blank; or 70 µL of control (100 µg mL^−1^ Procyanidin A2 in ethanol)/standard/samples were added to 210 µL DMAC solution (0.1 wt % in ethanol) in a well of 96-well plate. The mixture was shaken gently and incubated in the pre-heated chamber (25 °C). The microplate was read for 25 min.

The plate reader protocol was set to read the absorbance (640 nm) of each well in the plate every min for 30 min. The maximum absorbance readings were used for calculation.

### 3.9. Determination of Antioxidant Capacity

In this experiment, FRAP, DPPH, TEAC, and PCL (Photochemiluminescence assay) methods were used to measure, the antioxidant capacity.

#### 3.9.1. FRAP

The ferric reducing antioxidant power assay was performed as previously described by Benzie and Strain [59]. It is based on the reduction of the Fe^3+^-TPTZ complex to the ferrous form at low pH. This reduction is monitored by measuring the absorption change at 593 nm.

The reaction was carried out in a microtiter plate. 30 µL of distilled water and 10 µL properly diluted samples/standard were pipetted in a well of 96-well plate, then 200 µL FRAP reagent (10 volumes of 250 mM acetate buffer (pH 3.6), one volume of 20 mM ferric chloride solution and one volume of 10 mM tripyridyl-s-triazin (TPTZ) in 40 mM HCl) were added. The mixture was incubated at 37 °C and the absorbance was taken after 8 min at 593 nm.

The FRAP values was calculated and expressed as ascorbic acid equivalents per 100 g sample (fresh weight sour cherry/dried residue).

#### 3.9.2. DPPH

The DPPH free radical scavenging activity was measured using the method of Brand-Williams with modified as follows: 10 μL of appropriately diluted sample or Trolox solution (31.25, 62.5, 125, 250, 500, 750, and 1000 μM) and 50 μL distilled water was added to 190 μL of DPPH solution (0.1 mM in methanol) in a well of a 96-well plate. The mixture was shaken gently, incubated at 25 °C and the absorbance was taken after 30 min. The absorbance was measured at 517 nm, using the microplate reader. DPPH was expressed as milligrams of trolox equivalents (mg TE/100 g fresh weight sour cherry/dried residue) [26,60].

#### 3.9.3. TEAC

Determination of Trolox Equivalent Antioxidant Capacity (TEAC). This assay was performed as reported previously with slight modification. ABTS radical cations were prepared by mixing equal volumes of ABTS (7 mM in H_2_O) and potassium persulfate (4.9 mM in H_2_O), and the solution was left to stand in the dark for 12–16 h at room temperature; then the above solution was filtered and diluted with 80% ethanol to an absorbance of about 2 at 734 nm. 70 μL of 80% ethanol and 10 μL properly diluted samples/standard were pipetted in a well of 96-well plate, then 190 μL ABTS solution in a well of a 96-well plate, and the absorbance was recorded at 734 nm after 30 min of incubation at room temperature. Trolox was used as standard, and a standard calibration curve was obtained for Trolox at concentrations of 15.65, 31.25, 62.5, 125, 250, 500, and 1000 μM. The TEAC of samples was calculated from the standard curve of Trolox and expressed as gram of Trolox equivalents (TE) per 100 g of fresh weight sour cherry/dried residue (g TE/100 g) [61].

#### 3.9.4. Photochemiluminescence Assay (PLC)

This assay was described by Popov and Lewin, and distributed as a complete system under the name Photochem^®^ by Analytik Jena AG (Jena, Germany).

In the PCL assay, the photochemical generation of (O_2_^•−^) free radicals is combined with the sensitive detection by using chemiluminescence. The assay is initiated by optical excitation of photosensitizer (S), resulting in the generation of the superoxide radical anion.

S + hυ + O_2_→[S*O_2_]→S^•+^ + O_2_^•−^ (1)

There are two different protocols: ACW (water-soluble antioxidant capacity) and ACL (lipid-soluble antioxidant capacity) so both of the hydrophilic and the lipophilic antioxidants can be measured separately. These are standardized conditions, so the results are comparable to other assays. The antioxidant potential was assayed by means of the lag phase (ACW) or by means of the area under the curve (ACL) at different concentrations [51,52].

##### A. ACL

The lipophilic antioxidants were measured with the ACL kit. The reaction solutions were prepared by mixing 2.3 mL Reagent 1 (methanol), 200 μL Reagent 2 (buffer solution), 25 μL Reagent 3 (photosensitizer and detection reagent), and 0–30 μL of Reagent 4 (calibration standard for quantification of lipophilic antioxidants in Trolox equivalents) or 10 μL of sample (beverage diluted with Reagent 1) were mixed and measured. The detector measures the current proportion to the generated luminescence as a function of measurement time. The detector signal, monitored for 180 s. Results are expressed as mg equivalents of trolox per 100 g fresh weight sour cherry/dried residue [52].

##### B. ACW

The hydrophilic antioxidants were measured with the ACW kit. The reaction solutions were prepared by mixing 1.5 mL of Reagent 1 (buffer solution pH 10.5), 1 mL of Reagent 2 (reaction buffer), 25 μL Reagent 3 (photosensitizer and detection reagent) and 0–30 μL of Reagent 4 (calibration standard for quantification of water-soluble antioxidants in ascorbic acid equivalents) or 10 μL of sample (beverage diluted with Reagent 1). The detector measures the current proportion to the generated luminescence as a function of measurement time. The detector signal, monitored for 250 s, includes a lag phase in which no luminescence can be detected. When the antioxidants are exhausted, the amount of radicals in the sample increases until the detected signal reaches the maximum. The length of the lag phase increases in function of the amount of the antioxidants in the sample, and it is calculated by determining the first derivative and the maximum point of the detected curve. The interstion point of the slope of the straight line with the *x*-axis defines the lag time. Results are expressed as mg equivalents of ascorbic acid per 100 g fresh weight sour cherry/dried residue [51].

### 3.10. UHPLC Analysis

Measurements were carried out using CromasterUltraRs UHPLC, equipped with diode array detector, automatic sampler and Agillent OpenLAB software. The sample components were separated on a Phenomenex Kinetex column (2.6μ, XB.C18, 100A, 100 × 4.6 mm).

UHPLC running conditions consisted of the following linear gradient steps:

0 min solvent A 15%,

0–25 min solvent A to 30%,

25–30 min solvent A to 40%,

30–40 min solvent A to 50%.

Solvent A: MeOH; Solvent B: 3% HCOOH (Formic acid) in water.

Flow rate was 0.7 mL min^−1^ and oven temperature was kept at 25 °C. The anthocyanin content was analyzed quantitatively by comparison with the corresponding authentic standards. UV–vis detection was used at 535 nm wavelength for anthocyanins and 340 nm for flavonoid and phenolic compounds. The appropriate amounts of sour cherry extracts were measured and dissolved in solvent A. Injection volume was 10 μL.

### 3.11. UHPLC-MS Analysis

The UHPLC system (Dionex Ultimate 3000RS) was coupled to a Thermo Q Exactive Orbitrap mass spectrometer (Thermo Fisher Scientific Inc., Waltham, USA) equipped with an electrospray ionization source (ESI). The HPLC separation was achieved on a Themo Accucore C18 column (100 mm × 2.1 mm × 2.6 μm). Sampler and oven temperature were maintained at 25 °C, flow rate was 200 μL min^−1^. Eluent A was water containing 0.1% formic acid and eluent B was methanol containing 0.1% formic acid. The following gradient elution program was used: 0 min, 95% A; 0–3 min, 95% A; 3–43 min, →0% A; 43–61 min, 0% A; 61–62 min, →95% A; 62–70 min, 95% A. 2 μL of the samples were injected in every run. The Q Exactive hybrid quadrupole-orbitrap mass spectrometer was operated with the following parameters: capillary temperature 320 °C, spray voltage 4.0 kV in positive and 3.8 kV in negative ionization mode. The resolution was set to 35,000. The mass range scanned was 150–1500 *m*/*z*. The maximum injection time was 100 ms. The resolution was set to 17,500 in the cases of MS2 scans. The collision energy was 35 NCE. Sheath gas and aux gas flow rates were 32 and 7 arb, respectively. Xcalibur 4.0 (Thermo Fisher Scientific Inc., Waltham, USA) software was used to collect and analyze data.

### 3.12. Statistical Analysis

Data were expressed as means ± standard errors. Data were statistically analyzed using the SPSS statistical software, version 23. (SPSS Inc, Chicago, IL, USA). One-way analysis of variance (ANOVA) with Tukey’s honestly sgnificant difference test (homogeneity of variances) and Games–Howell significant difference test (not equal variances) were used to compare means among groups. The level of significance was set at *p* < 0.05.

## 4. Conclusions

It is known that different types of extractants can extract a variety of bioactive compounds. Accordingly, just one solvent is not sufficient to extract the total antioxidant compounds of foods, solvent combination is necessary, and the choice of suitable solvents is essential. As a result of our investigation, we found that the amounts and antioxidant properties of the dissolved compounds are not significantly different in case of solvent combination 1 and 2. However, Solvent combination 2 (methanol–acetone–water) can not be used in the food technology, only alcohol–water extraction is suitable.

The choice of the appropriate solvent is just the first step, the next is the determination of the exact antioxidant capacity. To do this you will need to find the most appropriate measurement method for the food type, because they contain different antioxidant compounds. FRAP method is good for the determination of water soluble, low molecular weight components. However, anthocyanins and associated flavonoids are only slightly soluble in water, so this method can not be measured these well. Although the solvent of DPPH and TEAC methods is ethanol, there are certain limitations that block measurement of the anthocyanins rich in sour cherry. For instance, because of the steric accessibility of DPPH• radical, the DPPH method can not adequately measure anthocyanin compounds [47], and in case of TEAC, the degree and position of hydroxylation and methoxylation in the B ring of anthocyanins, affects the stability and reactivity and thereby the antioxidant capacity [62]. However, the ACL and ACW methods are suitable to determine the antioxidant activity of various chemical components in SC.

## Figures and Tables

**Table 1:** Polyphenolic compounds identified by LC-MS in *Prunus cerasus* L. extracts.

No	RT [min]	Compound	Chemical Formula	Exact Mass (*m*/*z*)			Δ ppm	Fragment Ions (relative abundance, %)	Reference ^1^
				Measured [M + H]^+^	Measured [M − H]^-^	Calculated			
1 ^4^	10.37	Neochlorogenic acid	C16H18O9	355.10251		355.10291	−1.13	163.0391 (100); 145.0286 (11); 135.0443 (13)	[31]
2 ^4^	12.83	Coumaroylquinic acid isomer ^1^	C16H18O8		337.09293	337.09235	1.72	191.0553 (52); 163.0388 (100); 119.0487 (48)	[32]
3 ^4^	12.84	Procyanidin B isomer ^1^	C30H26O12		577.13531	577.13460	1.23	407.0771 (58); 289.0721 (60); 125.0229 (100)	[31,33]
4 ^4^	13.45	Coumaroylquinic acid isomer ^2^	C16H18O8		337.09317	337.09235	2.43	191.0555 (10); 163.0388 (100); 119.0487 (44)	[32]
5 ^3^	13.92	Procyanidin C isomer 1	C45H38O18		865.19922	865.19799	1.42	407.0771 (23); 289.0729 (30); 125.0229 (100)	[34]
6 ^4^	14.12	Catechin ^2^	C15H14O6		289.07196	289.07121	2.60	245.0819 (34); 151.0025 (63); 109.0280 (100)	[31,33]
7 ^3^	14.77	Procyanidin C isomer ^2^	C45H38O18		865.19910	865.19799	1.28	407.0774 (25); 289.0722 (26); 125.0230 (100)	[34]
8 ^4^	14.94	Chlorogenic acid ^2^	C16H18O9	355.10211		355.10291	−2.25	163.0390 (100); 145.0286 (11); 135.0443 (12)	[31,33]
9 ^4^	15.21	Feruloylquinic acid isomer ^1^	C17H20O9		367.10304	367.10291	0.35	193.0498 (100); 173.0445 (7); 134.0360 (65)	
10 ^4^	15.77	Procyanidin B isomer ^2^	C30H26O12		577.13519	577.13460	1.02	407.0768 (62); 289.0719 (64); 125.0230 (100)	[31,33]
11 ^3^	16.24	Chryptochlorogenic acid	C16H18O9	355.10223		355.10291	−1.92	163.0390 (100); 145.0285 (12); 135.0443 (12)	
12 ^4^	16.32	Coumaroylquinic acid isomer ^3^	C16H18O8		337.09201	337.09235	−1.01	191.0554 (16); 173.0444 (100); 163.0388 (19)	[32]
13 ^4^	16.42	Feruloylquinic acid isomer ^2^	C17H20O9		367.10301	367.10291	0.27	193.0497 (100); 173.0444 (85); 134.0362 (62)	
14 ^3^	17.23	Cyanidin-3-*O*-sophoroside	C27H30O16	611.16071		611.16122	−0.83	287.0552 (100); 213.0545 (4); 137.0226 (3)	[35]
15 ^3^	17.41	Procyanidin C isomer ^3^	C45H38O18		865.19835	865.19799	0.42	407.0762 (23); 289.0714 (27); 125.0230 (100)	[34]
16 ^4^	17.60	Cyanidin-3-*O*-glucoside ^2^	C21H20O11	449.10773		449.10839	−1.47	287.0552 (100); 213.0548 (3); 137.0229 (5)	[35]
17 ^3^	17.68	Epicatechin ^2^	C15H14O6		289.07175	289.07121	1.87	245.0818 (78); 151.0382 (31); 109.0281 (100)	[31,33]
18 ^4^	17.81	Cyanidin-3-*O*-(2^G^-glucosyl)-rutinoside	C33H40O20	757.21814		757.21912	−1.29	611.1639 (4); 287.0552 (100); 213.0547 (2)	[35]
19 ^4^	18.20	Coumaroylquinic acid isomer ^4^	C16H18O8		337.09311	337.09235	2.26	173.0444 (100); 163.0388 (23); 119.0487 (17)	[31,33]
20 ^3^	18.47	Cyanidin-3-*O*-rutinoside	C27H30O15	595.16626		595.16630	−0.07	449.1094 (4); 287.0552 (100); 213.0551 (2)	[35]
21 ^4^	18.54	Cyanidin-3-*O*-(2^G^-xylosyl)-rutinoside	C32H38O19	727.20795		727.20855	−0.83	581.1515 (3); 287.0553 (100); 213.0545 (2)	[35]
22 ^4^	18.80	Pelargonidin-3-*O*-(2^G^-glucosyl)rutinoside	C33H40O19	741.22491		741.22420	0.96	271.0602 (100)	[35]
23 ^3^	19.41	Cinchonain I isomer ^1^	C24H20O9		451.10318	451.10291	0.60	341.0666 (100); 217.0137 (33)	
24 ^3^	19.45	Pelargonidin-3-*O*-rutinoside	C27H30O14	579.17096		579.17138	−0.73	433.1135 (4); 271.0603 (100);	[35]
25 ^4^	20.05	Peonidin-3-*O*-rutinoside	C28H32O15	609.18152		609.18195	−0.71	463.1237 (4); 301.0708 (100); 286.0474 (14)	[35]
26 ^4^	20.35	Cyanidin-*O*-pentoside	C20H18O10	419.09750		419.09783	−0.79	287.0554 (100)	
27 ^3^	20.36	Quercetin-*O*-(hexosyl)rutinoside	C33H40O21		771.19940	771.19839	1.31	300.0276 (100); 271.0248 (40); 255.0298 (19)	
28 ^4^	20.76	Quercetin-*O*-(hexosyl)hexoside isomer ^1^	C27H30O17		625.14056	625.14048	0.13	300.0276 (100); 271.0251 (37); 255.0305 (20)	
29 ^3^	20.96	Quercetin-di-*O*-hexoside	C27H30O17		625.14111	625.14048	1.01	463.0888 (48); 301.0356 (70); 300.0277 (100)	
30 ^4^	21.01	Procyanidin B isomer ^3^	C30H26O12		577.13605	577.13460	2.51	407.0768 (31); 289.0724 (53); 125.0229 (100)	[31,33]
31 ^3^	21.09	Quercetin-*O*-rutinoside-*O*-glucoside	C33H40O21		771,19904	771.19839	0.84	609.1465 (89); 301.0355 (90); 300.0277 (100)	[34]
32 ^4^	21.44	Naringenin chalcone-*O*-hexoside	C21H22O10		433.11380	433.11348	0.74	271.0613 (100); 151.0024 (59); 119.0488 (22)	
33 ^3^	21.81	Cinchonain I isomer ^2^	C24H20O9		451.10123	451.10291	−3.72	341.0667 (100); 217.0135 (36)	
34 ^4^	22.27	Quercetin-*O*-(hexosyl)hexoside isomer ^2^	C27H30O17		625.14001	625.14048	−0.75	300.0277 (100); 271.0244 (33); 255.0288 (18)	
35 ^3^	22.81	Cinchonain I isomer ^3^	C24H20O9		451.10248	451.10291	−0.95	341.0667 (100); 217.0138 (39)	
36 ^4^	22.88	Di-*O*-caffeoylquinic acid	C25H24O12		515.11914	515.11896	0.35	353.0879 (60); 191.0553 (100); 179.0339 (62)	
37 ^4^	22.99	Prunin	C21H22O10		433.11389	433.11348	0.95	271.0612 (100); 151.0024 (41); 119.0487 (26)	[36]
38 ^4^	23.55	Isoquercitrin ^2^	C21H20O12		463.08810	463.08765	0.97	301.0354 (43); 300.0276 (100); 271.0249 (37)	[31,33]
39 ^4^	23.63	Rutin ^2^	C27H30O16	611.16071		611.16122	−0.83	465.1029 (3); 303.0500 (100); 85.0289 (16)	[31,33]
40 ^3^	23.74	Dihydroxy(iso)flavone-*C*-glucoside	C21H20O9	417.11816		417.11856	−0.96	399.1080 (33); 381.0978 (25); 297.0760 (100)	
41 ^3^	25.34	Astragalin	C21H20O11		447.09348	447.09274	1.66	285.0406 (66); 284.0328 (100); 255.0297 (86)	
42 ^4^	25.47	Nicotiflorin	C27H30O15		593.15094	593.15065	0.49	285.0406 (100); 284.0328 (73); 255.0298 (42)	
43 ^3^	25.51	Cinchonain I isomer ^4^	C24H20O9		451.10400	451.10291	1.35	341.06747 (100); 217.01355 (48)	
44 ^4^	25.83	Narcissin	C28H32O16		623.16132	623.16122	0.16	315.0512 (100); 314.0435 (44); 299.0197 (40)	[31,33]
45 ^3^	27.30	Quercetin-3-*O*-(4-coumaroyl)glucoside	C30H26O14		609.12531	609.12444	1.43	463.0896 (41); 300.0279 (100); 271.0247 25)	[31,33]
46 ^4^	27.89	Naringenin ^2^	C15H12O5		271.06122	271.06065	2.10	177.0182 (17); 151.0024 (100); 119.0488 (80)	[31,33]

**^1^** Identified compounds in *Prunus cerasus L*. in the literature. **^2^** Confirmed by standard. **^3^** Detected only in the ethanol/water extract (1/A + 1/B). **^4^** Detected in both extracts (1/A + 1/B and 2/a + 2/B).

## Supplementary Materials

The following are available.

## Dedication

This study is dedicated to Professor Sándor Biró on the occasion of his 70th birthday, and we honor his undisputed merits in the field of the microbial genetics.

## References

[1] Olden E.J., Nybom N. (1968). On the origin of the *Prunus cerasus* L.. Hereditas.

[2] Wang H., Nair M.G., Iezzoni A.F., Strasburg G.M., Booren A.M., Gray J.I. (1997). Quantification and Characterization of Anthocyanins in Balaton Tart Cherries. J. Agric. Food Chem..

[3] Myhrstad M.C., Carlsen H., Nordstrom O., Blomhoff R., Moskaug J.O. (2002). Flavonoids increase the cellular glutathione level by transactivation of the g-glutamylcysteine synthetase catalytical subunit promoter. Free Radic. Biol. Med..

[4] Moskaug J.O., Carlsen H., Myhrstad M.C.W., Blomhoff R. (2005). Polyphenols and glutathione synthesis regulation. Am. J. Clin. Nutr..

[5] Gutierrez R.M. (2012). Effect of the hexane extract of Piper auritum on insulin release from beta-cell and oxidative stress in streptozotocin-induced diabetic rat. Pharmacogn. Mag..

[6] Mane C., Loonis M., Juhel C., Dufour C., Malien-Aubert C. (2011). Food grade lingonberry extract: Polyphenolic composition and in vivo protective effect against oxidative stress. J. Agric. Food Chem..

[7] Martin M.A., Fernández-Millán E., Ramos S., Bravo L., Goya L. (2014). Cocoa flavonoid epicatechin protects pancreatic beta cell viability and function against oxidative stress. Mol. Nutr. Food Res..

[8] Youdim K.A., Martin A., Joseph J.A. (2000). Incorporation of the elderberry anthocya- nins by EC increases protection against oxidative stress. Free Radic. Biol. Med..

[9] Ciz M., Denev P., Kratchanova M., Vasicek O., Ambrozova G., Lojek A. (2012). Flavonoids Inhibit the Respiratory Burst of Neutrophils in Mammals. Oxid. Med. Cell. Longev..

[10] Cassidy A., O’Reilly E.J., Kay C., Sampson L., Franz M., Forman J.P., Curhan G., Rimm E.B. (2011). Habitual intake of flavonoid subclasses and incident hypertension in adults. Am. J. Clin. Nutr..

[11] Xu J.W., Ikeda K., Yamori Y. (2004). Cyanidin-3-glucoside regulates phosphorylation of endothelial nitric oxide synthase. FEBS Lett..

[12] Watson R.R., Preedy V.R., Zibadi S. (2014). Polyphenols in Human Health and Disease.

[13] Guo H., Ling W., Wang Q., Liu C., Hu Y., Xia M. (2008). Cyanidin 3-glucoside protects 3T3-L1 adipocytes against H_2_O_2_- or TNF-α-induced insulin resistance by inhibiting c-Jun NH2-terminal kinase activation. Biochem. Pharmacol..

[14] Tang Y., Zhang B., Li X., Chen P.X., Zhang H., Liu R., Tsao R. (2016). Bound Phenolics of Quinoa Seeds Released by Acid, Alkaline, and Enzymatic Treatments and Their Antioxidant and α-Glucosidase and Pancreatic Lipase Inhibitory Effects. J. Agric. Food Chem..

[15] Shahidi F., Yeo J.D. (2016). Insoluble-Bound Phenolics in Food. Molecules.

[16] Pérez-Jiménez J., Arranz S., Tabernero M., Díaz-Rubio M.E., Serrano J., Goñi I., Saura-Calixto F. (2008). Updated methodology to determine antioxidant capacity in plant foods, oils and beverages: Extraction, measurement and expression of results. Food Res. Int..

[17] Andreasen M.F., Kroon P.A., Williamson G., Garcia-Conesa M.T. (2001). Esterase Activity Able to Hydrolyze Dietary Antioxidant Hydroxycinnamates Is Distributed along the Intestine of Mammals. J. Agric. Food Chem..

[18] Pérez-Jiménez J., Díaz-Rubio M.E., Saura-Calixto F. (2013). Non-extractable polyphenols, a major dietary antioxidant: Occurrence, metabolic fate and health effects. Nutr. Res. Rev..

[19] Pérez-Jiménez J., Saura-Calixto F. (2015). Macromolecular antioxidants or non-extractable polyphenols in fruit and vegetables: Intake in four European countries. Food Res. Int..

[20] Kristl J., Slekovec M., Tojnko S., Unuk T. (2011). Extractable antioxidants and non-extractable phenolics in the total antioxidant activity of selected plum cultivars (*Prunus domestica* L.): Evolution during on-tree ripening. Food Chem..

[21] Gómez-García R., Martínez-Ávila G.C.G., Aguilar C.N. (2012). Enzyme-assisted extraction of antioxidative phenolics from grape (*Vitis vinifera* L.) residues. 3 Biotech.

[22] Guo L. (2017). Enzymatic hydrolysis of lotus rhizome starch using alpha-amylase and glucoamylase. J. Food Nutr. Res..

[23] Anokwuru C., Sigidi M., Boukandou M., Tshisikhawe P., Traore A., Potgieter N. (2018). Antioxidant Activity and Spectroscopic Characteristics of Extractable and Non-Extractable Phenolics from Terminalia sericea Burch. ex DC. Molecules.

[24] Wang H., Cao G. (1997). Oxygen radicals absorbing capacity of anthocyanins. J. Agric. Food Chem..

[25] Frankel E.N., Meyer A.S. (2000). The problems of using one-dimensional methods to evaluate multifunctional food and biological antioxidants. J. Sci. Agric..

[26] Brand-Williams W., Cuvelier M.E., Berset C. (1995). Use of a free radical method to evaluate antioxidant activity. Food Sci. Technol..

[27] Sanchez-Moreno C., Larrauri J.A., Saura-Calixto F. (1998). A procedure to measure the antiradical efficiency of polyphenols. J. Sci. Food Agric..

[28] Saura-Calixto F., Goñi I. (2006). Antioxidant capacity of the Spanish Mediterranean diet. Food Chem..

[29] Homoki J.R., Nemes A., Fazekas E., Gyémánt G., Balogh P., Gál F., Al-Asri J., Mortier J., Wolber G., Babinszky L. (2016). Anthocyanin composition, antioxidant efficiency, and a-amylase inhibitor activity of different Hungarian sour cherry varieties (*Prunus cerasus* L.). Food Chem..

[30] Chaovanalikit A., Wrolstad R.E. (2004). Anthocyanin and polyphenolic composition of fresh and processed cherries. J. Food Sci..

[31] Levaj B., Dragović-Uzelac V., Delonga K., Ganić K.K., Banović M., Kovačević D.B. (2010). Polyphenols and volatiles in fruits of two sour cherry cultivars, some berry fruits and their jams. Food Technol. Biotechnol..

[32] Han J.H., Lee H.J., Cho M.R., Chang N., Kim Y., Oh S.Y., Kang M.H. (2014). Total antioxidant capacity of the Korean diet. Nutr. Res. Pract..

[33] Bonerz D., Würth K., Dietrich H., Will F. (2007). Analytical characterization and the impact of ageing on anthocyanin composition and degradation in juices from five sour cherry cultivars. Eur. Food Res. Technol..

[34] Wojdyło A., Nowicka P., Laskowski P., Oszmiański J. (2014). Evaluation of sour cherry (*Prunus cerasus* L.) fruits for their polyphenol content, antioxidant properties, and nutritional components. J. Agric. Food Chem..

[35] Jakobek L., Seruga M., Seruga B., Novak I., Medvicovic-Kosanovic M. (2009). Phenolic compound composition and antioxidant activity of fruits of Rubus and Prunus species from Croatia. Int. J. Food Sci. Technol..

[36] Toydemir G., Capanoglu E., Gomez-Roldan M.V., de Vos R.C.H., Boyacioglu D., Hall R.D., Beekwilder M.J. (2013). Industrial processing effects on phenolic compounds in sour cherry (*Prunus cerasus* L.) fruit. Food Res. Int..

[37] Arteel G.E., Sies H. (1999). Protection against peroxinitrite by cocoa polyphenol oligomers. FEBS Lett..

[38] Lunder T.L., Huang M.T., Ho C.T., Lee C.Y. (1992). Catechins of green tea: Antioxidant activity. Phenolic Compounds in Food and Their Effects on Health II.

[39] Pannala A.S., Chan T.S., O’Brien P.J., Rice-Evans C.A. (2001). Flavonoid B-ring chemistry and antioxidant activity: Fast reaction kinetics. Biochem. Biophys. Res. Commun..

[40] Rice-Evans C.A., Packer L. (1997). Flavonoids in Health and Disease.

[41] Gonzales-Manzano S., Santos-Buelga C., Perez-Alonso J.J., Rivas-Gonzalo J.C., Escribano-Bailon M.T. (2006). Characterization of the mean degree of polymerization of proanthocyanidins in red wines using Liquid Chromatography-Mass Spectrometry (LC-MS). J. Agric. Food Chem..

[42] Hagerman A.E., Riedl K.M., Jones G.A., Sovik K.N., Ritchard N.T., Hartzfeld P.W., Riechel T.L. (1998). High molecular weight plant polyphenolics (tannins) as biological antioxidants. J. Agric. Food Chem..

[43] Shi J., Yu J., Pohorly J.E., Kakuda Y. (2003). Polyphenolics in grape seeds-biochemistry and functionality. J. Med. Food.

[44] Khanal R.C., Howard L.R., Prior R.L. (2009). Procyanidin content of grape seed and pomace, and total anthocyanin content of grape pomace as affected by extrusion processing. J. Food Sci..

[45] Manach C., Williamson G., Morand C., Scalbert A., Remesy C. (2005). Bioavailability and bioefficacy of polyphenols in humans. I. Review of 97 bioavailability studies. Am. J. Clin. Nutr..

[46] Capanoglu E., Boyacioglu D., de Vos R.C.H., Hall R.D., Beekwilder J. (2011). Procyanidins in fruit from Sour cherry (*Prunus cerasus*) differ strongly in chainlength from those in Laurel cherry (*Prunus lauracerasus*) and Cornelian cherry (*Cornus mas*). J. Berry Res..

[47] Boligon A.A., Machado M.M., Athayde M.L. (2014). Technical evaluation of antioxidant activity. Med. Chem..

[48] Prior R.L., Cao G. (2000). Analysis of botanicals and dietary supplements for antioxidant capacity: A review. J. AOAC Int..

[49] Huang D., Ou B., Prior R.L. (2005). The chemistry behind antioxidants capacity assays. J. Agric. Food Chem..

[50] Litwinienko G., Ingold K.U. (2003). Abnormal solvent effects on hydrogen atom abstractions. 1. The reactions of phenols with 2,2-diphenyl-1-picrylhydrazyl (dpph•) in alcohols. J. Org. Chem..

[51] Popov I.N., Lewin G. (1994). Photochemiluminescent detection of antiradical activity. 2. Testing nonenzymic water-soluble antioxidants. Free Radic. Biol. Med..

[52] Popov I.N., Lewin G. (1996). Photochemiluminescent detection of antiradical activity; IV: Testing of lipid-soluble antioxidants. J. Biochem. Biophys. Methods.

[53] Kim D., Heo H.J., Yang H.S., Lee C.Y. (2005). Sweet and sour cherry phenolics and their protective effects on neuronal cells. J. Agric. Food Chem..

[54] Hartzfeld P.W., Forkner R., Hunter D.M., Hagerman A.E. (2002). Determination of hydrolysable tannins (gallotannins and ellagitannins) after reaction with potassium iodate. J. Agric. Food Chem..

[55] Porter L., Hrstich L., Chan B. (1985). The conversion of procyanidins and prodelphinidins to cyaniding and delphinidin. Phytochemistry.

[56] Reed J., McDowell R.E., Van Soest P.J., Horvarth P.J. (1982). Condensed tannins: A factor limiting the use of cassava forage. J. Sci. Food Agric..

[57] Singleton V.L., Orthofer R., Lamuela-Raventós R.M. (1999). Analysis of total phenols and other oxidation substrates and antioxidants by means of folin-ciocalteu reagent. Methods Enzymol..

[58] Prior R.L., Fan E., Ji H., Howell A., Nio C., Payne M.J., Reed J. (2010). Multi-laboratory validation of a standard method for quantifying proanthocyanidins in cranberry powders. J. Sci. Food Agric..

[59] Benzie I.F., Strain J.J. (1996). The ferric reducing ability of plasma (FRAP) as a measure of “antioxidant power”: The FRAP assay. Anal. Biochem..

[60] Blois M.S. (1958). Antioxidant determinations by the use of a stable free radical. Nature.

[61] Miller N.J., Rice-Evans C.A., Davies M.J., Gopinathan V., Milner A. (1993). A novel method for measuring antioxidant capacity and its application to monitoring the antioxidant status in premature neonates. Clin. Sci..

[62] Montoro P., Tuberoso C.I., Piacente S., Perrone A., De Feo V., Cabras P., Pizza C. (2006). Stability and antioxidant activity of polyphenols in extracts of Myrtus communis L. berries used for the preparation of myrtle liqueur. J. Pharm. Biomed. Anal..

