# Supplementary material for: Determination of Flavonoid and Proanthocyanidin Profile of Hungarian Sour Cherry
Source: Molecules. 2018 Dec 11;23(12):3278. doi: 10.3390/molecules23123278 (PMC6321385; doi:10.3390/molecules23123278)
Supplement: Supplementary file 1 [file molecules-23-03278-s001.pdf]

Table captions

Supplements

Supplement 1

Sample	1/AS+1/BS			2/AS+2/BS			1/AS			1/BS			2/AS			2/BS		
Method	Mean	Std. Error	P-value	Mean	Std. Error	P-value	Mean	Std. Error	P-value	Mean	Std. Error	P-value	Mean	Std. Error	P-value	Mean	Std. Error	P-value
ACL	0.239	0.006	**b**	0.232	0.004	**b**	0.151	0.001	**a**	0.030	0.002	**a**	0,105	0,002	**a**	0,052	0,001	**b**
TEAC	0.189	0.002	**c**	0.171	0.002	**c**	0.073	0.001	**d**	0.023	0.000	**bc**	0,061	0,004	**bc**	0,020	0,003	**c**
DPPH	0.168	0.004	**d**	0.118	0.007	**d**	0.075	0.001	**d**	0.015	0.001	**de**	0,059	0,006	**c**	0,028	0,000	**c**
ACW	0.268	0.005	**a**	0.294	0.007	**a**	0.097	0.001	**c**	0.026	0.003	**ab**	0,124	0,018	**a**	0,029	0,003	**c**
FRAP	0.185	0.005	**cd**	0.188	0.010	**c**	0.116	0.000	**b**	0.018	0.000	**cd**	0,098	0,008	**ab**	0,088	0,012	**a**
TPC	0.053	0.003	**e**	0.070	0.001	**e**	0.052	0.001	**e**	0.011	0.000	**e**	0,041	0,004	**c**	0,018	0,000	**c**

Supplement 2

Sample	1/RH			2/RH			1/RK			2/RK			1/RPR			1/RA		
Method	Mean	Std. Error	P-value	Mean	Std. Error	P-value	Mean	Std. Error	P-value	Mean	Std. Error	P-value	Mean	Std. Error	P-value	Mean	Std. Error	P-value
ACL	5,523924	0,206837	**b**	4,626273	0,089568	**b**	5,136208	0,281042	**bc**	3,376704	0,172545	**b**	0,329652	0,005146	**a**	0,297342	0,009064	**c**
TEAC	0,814408	0,01602	**e**	0,655186	0,024693	**e**	6,089197	0,087278	**b**	5,577601	0,081766	**b**	0,214855	0,129988	**a**	0,345807	0,005434	**b**
DPPH	8,414576	0,18805	**a**	8,14816	0,157792	**a**	250,02	2,510623	**a**	247,7094	4,743269	**a**	0,271361	0,003104	**a**	0,159186	0,012789	**d**
ACW	2,239381	0,024139	**c**	2,222443	0,028895	**c**							0,164468	0,001113	**a**	0,087363	0,002353	**e**
FRAP	1,302172	0,025742	**d**	0,404671	0,015198	**e**	1,287592	0,060399	**c**	1,22635	0,008982	**b**	0,222895	0,006201	**a**	0,14819	0,012164	**d**
TPC	2,17133	0,07447	**c**	1,412789	0,099435	**d**	3,126382	0,249972	**bc**	3,662156	0,250009	**b**	0,182549	0,000621	**a**	1,249431	0,015739	**a**

Sample	1/RL			2/RPR			2/RA			2/RL			1/RPE			2/RPE		
Method	Mean	Std. Error	P-value	Mean	Std. Error	P-value	Mean	Std. Error	P-value	Mean	Std. Error	P-value	Mean	Std. Error	P-value	Mean	Std. Error	P-value
ACL	0,296789	0,001204	**d**	0,223075	0,012261	**a**	0,188443	0,014859	**a**	0,381569	0,003655	**d**	0,300673	0,005271	**a**	0,298767	0,002766	**a**

TEAC	2,632729	0,034454	**b**	0,091801	0,001142	**c**	0,074066	0,002684	**c**	2,589332	0,014251	**b**	0,293242	0,009712	**a**	0,310956	0,004563	**a**
DPPH				0,146715	0,00561	**b**	0,092937	0,005971	**bc**				0,146153	0,005179	**c**	0,125941	0,001698	**c**
ACW	0,265097	0,001244	**d**	0,1426	0,001015	**b**	0,087396	0,000733	**bc**	0,336876	0,000551	**d**	0,105441	0,002593	**d**	0,109604	0,004282	**d**
FRAP	1,485204	0,015161	**c**	0,141947	0,003666	**b**	0,071692	0,000851	**c**	1,470293	0,006853	**c**	0,194373	0,002599	**b**	0,187999	0,00318	**b**
TPC	3,601912	0,042691	**a**	0,132874	0,002326	**b**	0,104849	0,001015	**b**	3,70971	0,037335	**a**	0,191527	0,00204	**b**	0,190108	0,004256	**b**

Supplement 3 Comparison of antioxidant capacity assays

Method	Advantages	Disadvantages
FRAP	• It is simple, speedy, inexpensive, and robust does not required specialized equipment [1, 2].<br><br>• It can be performed using automated, semiautomated, or manual methods [1].<br><br>• It was originally applied to plasma but has been extended to other biological fluids, foods, plant extracts and juices [3].<br><br>• This method has been adapted to 96 well microplate reader, giving better reproducibility and higher sample throughput.	• TPTZ• radical don't exist in biological systems.<br><br>• Not all antioxidants are able to reduce Fe, antioxidants that act by H atom transfer are not detected, particularly SH group containing antioxidants like thiols, such as glutathione and proteins [2, 4, 5].<br><br>• The introduction of Fe may result in the generation of additional free radicals [2].<br><br>• This method essentially provides the stoichiometry of antioxidants, which for instance has been determined as two for ascorbic acid, uric acid and α-tocopherol, but about 4 for bilirubin and zero for albumin [6].<br><br>• In the cases, the reaction is not complete after 4 minutes, so the result of this test depends on the reaction time [6].<br><br>• The reaction is nonspecific, and any compound with a suitable redox potential will drive FeIII-TPTZ reduction [7].
DPPH	• The DPPH• radical is stable, commercially available, and does not have to be generated before assay (like $ABTS^{\bullet+}$) [2].<br><br>• It is considered an easy and useful spectrophotometric method with regard to screening/measuring the antioxidant capacity of both pure compounds and complex samples [2].<br><br>• It is a rapid, simple, inexpensive and widely used method to measure the ability of compounds to act as free radical scavengers or hydrogen donors, and to evaluate antioxidant activity of foods. It can also be used to quantify antioxidants in complex biological systems, for solid or liquid samples [8, 9].<br><br>• This method is easy and applies to measure the overall antioxidant capacity and the free radical scavenging activity of fruit and vegetable juices [8, 9].<br><br>• It has been adapted to 96 well microplate reader, giving better reproducibility and higher sample throughput.	• DPPH• radical don't exist in biological systems.<br><br>• The linear relationship between the rate constants with DPPH• and ROO• only holds in aprotic solvents, whereas in polar protic solvents (i.e. ethanol or methanol: DPPH• is insoluble in water) the reaction between DPPH• and phenols is strongly accelerated by a stepwise proton-transfer electron-transfer mechanism (named SPLET) [10].<br><br>• Using MeOH as solvent, acidic phenols react much faster than expected with DPPH•, which would subvert the order of reactivity with respect to their actual antioxidant activity [10].<br><br>• The steric accessibility of DPPH• radical is a major determinant of the reaction. The small molecules that have better access to the radical site have relatively higher antioxidant capacity. In case of many large antioxidant compounds that react quickly with peroxyl radicals may react slowly or may even be inert in this assay [2].<br><br>• The spectrophotometric measurements can be affected by compounds, such as carotenoids, that absorb at the wavelength of determination as well as by the turbidity of the sample [11].<br><br>• The DPPH• assay is not suitable for measuring the antioxidant capacity of plasma, because proteins are precipitated in the alcoholic reaction medium [2].

		• The DPPH• scavenging reaction is timeconsuming and it may take 20 min up to 6 h [12].
TEAC	• The method is rapid and can be used over a wide range of pH values, which is useful to study the effect of pH on antioxidant mechanisms [2].<br><br>• The ABTS•+ radical is stable and soluble in water and organic solvents, enabling the determination of antioxidant capacity of both hydrophilic and lipophilic compounds/ samples/ body fluids [2].<br><br>• It also has good repeatability and is simple to perform; hence, it is widely reported [2].<br><br>• TEAC assay is operationally simple; it has been used in many research laboratories for studying antioxidant capacity. TEAC values of many compounds and food samples have been reported. ABTS•+ reacts rapidly with antioxidants, typically within 30 min [2].<br><br>• It has been adapted to 96 well microplate reader, giving better reproducibility and higher sample throughput.	• ABTS+• radical don't exist in biological systems.<br><br>• This test does not distinguish between kinetics of radical trapping and stoichiometry, and the outcome may depend on the time chosen for reading the absorbance [13].<br><br>• By following the time course of the reaction for longer periods, Perez-Jimenez and Saura-Calixto have shown that α-tocopherol and ascorbic acid have a stoichiometry slightly larger than $n = 2$, that is the number expected from the "usual" radical trapping mechanism of phenolic antioxidants, while other inhibitors have significantly larger values (resveratrol: $n = 6$; quercetin: $n = 7$) [14].<br><br>• On the other hand, the time required to reach the stationary concentration, which inversely depends on the rate constant for the reaction between antioxidants and ABTS+•, widely varies among antioxidants, being smaller for α-tocopherol and ascorbic acid [14].<br><br>• An important limitation of this assay is that ABTS+• is a radical cation while the peroxyl radical is neutral, so antioxidants react with ABTS+• by an electron transfer mechanism, whereas with peroxyl radicals they react by formal H atom transfer [15].<br><br>• However, as the results obtained for samples are related to an antioxidant standard compound that shows different kinetic behavior, the results provided by this assay are dependent on the time of analysis. ABTS assay is frequently used by the food industry and agricultural researchers to measure the antioxidant capacities of foods [2, 16].<br><br>• Disadvantages of TEAC assay: ABTS•+ used in TEAC assay is not found in mammalian biology and thus represents a "non physiological" radical source. Thermodynamically, a compound can reduce ABTS•+ if it has a redox potential lower than that of ABTS (0.68 V) [11, 17].<br><br>• Many phenolic compounds have low redox potentials and thus react with ABTS•+. The TEAC reaction may not be the same for slow reactions, and it may take a long time to get the endpoint. Thus, by using an endpoint of short duration (4 or 6 min), one may be reading before the reaction is finished and result in lowered TEAC values [2].<br><br>• The degree and position of hydroxylation and methoxylation in the B ring of anthocyanins, affects the stability and reactivity and thereby the antioxidant capacity [18].

PLC	• Superoxide radical exist in biological systems.<br><br>• Time- and cost-effective system for the determination of the integral antioxidative capacity toward $O2^{\bullet -}$ with ACW and ACL ready-to-use reagent kits [19].<br>• a sample may require, at most, 3 min for analysis [19].<br><br>• The conditions are standardized, so the results are comparable to other assays [19].<br><br>• The PHOTOCHEM® device only measures the antioxidative effectiveness of test samples against the PCL resulting from one free radical, namely $O2^{\bullet -}$ [20].	• PCL measurements are collected at nonphysiological pH values; hence, it is difficult to transfer the results of foodstuffs by this assay to the physiological environment of the human body [20].<br><br>• Only one sample can be measured at a time, it is not, in its present configuration adaptable to a high-throughput assay system [19].

References:

1. Benzie, I.F.; Strain, J.J. Ferric reducing antioxidant power assay; direct measure of total antioxidant activity of biological fluids and modified version for simultaneous measurement of total antioxidant power and ascorbic acid concentration. *Methods Enzymol*. **1999**, *299*: 15-27. PMID: 9916193.
2. Boligon, A.A.; Machado, M.M.; Athayde, M.L. Technical evaluation of antioxidant activity. *Med. Chem*. **2014**, *4*: 517-522. DOI: 10.4172/2161-0444.1000188.
3. MagalhÃ£es, L.M.; Segundo, M.A.; Reis, S.; Lima, J.L. Methodological aspects about in vitro evaluation of antioxidant properties. *Anal. Chim. Acta*. **2008**, *613*: 1-19. DOI: 10.1016/j.aca.2008.02.047. PMID: 18374697.
4. Prior, R.L; Cao, G. Analysis of botanicals and dietary supplements for antioxidant capacity; a review, *J. AOAC Int.* **2000**, *83*: 950 – 955. PMID: 10995120.
5. Huang, D.; Ou, B.; Prior, R.L. The chemistry behind antioxidants capacity assays. *J. Agric. Food Chem*. **2005**, *53*: 1841-1856. DOI: 10.1021/jf030723c. PMID: 15769103.
6. Amorati, R.; Valgimigli, L. Advantages and limitations of common testing methods for antioxidants. *Free Radic. Res*. **2015**, *49*: 633–649. DOI: 10.3109/10715762.2014.996146.
7. Benzie, I.F.; Strain, J.J. The ferric reducing ability of plasma (FRAP) as a measure of "antioxidant power": the FRAP assay. *Anal. Biochem*. **1996**, *239*:70-76. DOI: 10.1006/abio.1996.0292. PMID: 8660627.
8. Prakash, A. Antioxidant activity. *Med. Lab. Anal. Prog*. **2001**, *19*: 1–6.
9. Sendra, J.M.; Sentandreu, E.; Navarro, J.L. Reduction kinetics of the free stable radical 2, 2-diphenyl-1-picrylhydrazyl (DPPH•) for determination of the antiradical activity of citrus juices. *Eur. Food Res. Technol*. **2006**, *223*:615–624. DOI: 10.1007/s00217-005-0243-3.
10. Litwinienko, G.; Ingold, K.U. Abnormal solvent effects on hydrogen atom abstractions. 1. The reactions of phenols with 2,2-diphenyl-1-picrylhydrazyl (dpph•) in alcohols. *J. Org. Chem*. **2003**, *68*:3433-3438. DOI: 10.1021/jo026917t. PMID: 12713343.
11. Apak, R.; Gorinstein, S.; Bohm, V.; Schaich, K.M.; Özyürek, M.; Güçlü, K. Methods of measurement and evaluation of natural antioxidant capacity/activity (IUPAC

Technical Report). *Pure Appl. Chem*. **2013**, *85*: 957-998. DOI: 10.1351/PAC-REP-12-07-15.

12. Magalhães, L.M.; Segundo, M.A.; Reis, S.; Lima, J.L. Methodological aspects about in vitro evaluation of antioxidant properties. *Anal. Chim. Acta* **2006**, *558*: 310-318. DOI: 10.1016/j.aca.2008.02.047. PMID: 18374697.
13. Tian, X.; Schaich, K.M. Effects of molecular structure on kinetics and dynamics of the Trolox Equivalent Antioxidant Capacity assay with ABTS+•. *J. Agric. Food Chem.* **2013**, *61*:5511−5519. DOI: 10.1021/jf4010725. PMID: 23659464.
14. Perez-Jimenez, J.; Saura-Calixto, F. Anti-oxidant capacity of dietary polyphenols determined by ABTS assay: a kinetic expression of the results. *Int. J. Food Sci. Technol*. **2008**, *48*: 185-191. DOI: 10.1111/j.1365-2621.2006.01425.x.
15. Amorati, R.; Menichetti, S.; Viglianisi, C.; Foti, M.C. Proton–electron transfer pathways in the reactions of peroxyl and dpph radicals with hydrogen-bonded phenols. *Chem. Commun. (Camb).* **2012**, *48*: 11904–11906. DOI: 10.1039/c2cc36531f. PMID: 23125978.
16. Alam, M.N.; Bristi, N.J.; Rafiquzzaman, M. Review on in vivo and in vitro methods evaluation of antioxidant activity. *Saudi Pharm. J.* **2013**, *21*: 143-152. DOI: 10.1016/j.jsps.2012.05.002.
17. Antolovich, M.; Prenzler, P.D.; Patsalides, E.; McDonald, S.; Robards, K. Methods for testing antioxidant activity. *Analyst* **2002**, *127*: 183-198. PMID: 11827390.
18. Montoro, P.; Tuberoso, C.I.; Piacente, S.; Perrone, A.; De Feo, V.; Cabras, P.; Pizza C. Stability and antioxidant activity of polyphenols in extracts of Myrtus communis L. berries used for the preparation of myrtle liqueur. *J. Pharm.. Biomed. Anal*. **2006**, *41*: 1614-1619. DOI: 10.1016/j.jpba.2006.02.018. PMID: 16554139.
19. Schlesier, K.; Harwat, M.; Böhm, V.; Bitsch, R. Assessment of antioxidant activity by using different in vitro methods. *Free Radic. Res*. **2002**, *36*, 177-187. DOI: 10.1080/10715760290006411. PMID: 11999386.
20. Pegg, R.B.; Amarowicz, R.; Naczk, M.; Shahidi, F. Chapter 11: PHOTOCHEM® for Determination of Antioxidant Capacity of Plant Extracts. In *ACS Symposium Series*, 2007, Volume 956, pp 140–158; eISBN: 9780841220744. DOI:10.1021/bk-2007-0956.ch011.
